# Preliminary Characterization of Monofloral *Helianthus annuus* L. Bee Pollen Using Advanced Analytical Techniques: A Comparative Study With Polyfloral Pollen

**DOI:** 10.1002/fsn3.71016

**Published:** 2025-09-26

**Authors:** Nazlıcan Yıldırım, Yusuf Can Gerçek

**Affiliations:** ^1^ Institute of Graduate Studies in Sciences Istanbul University Istanbul Türkiye; ^2^ Department of Biology, Faculty of Science Istanbul University Istanbul Türkiye

## Abstract

In recent years, there has been an increased interest in the characterization of monofloral bee pollen of different botanical origins as it offers a more homogeneous and traceable component profile compared to polyfloral bee pollen. In this study, we aimed to verify the botanical origin of monofloral *Helianthus annuus* (sunflower) pollen and polyfloral bee pollen samples collected from the same geographical region and to evaluate their metabolomic profiles comparatively. The botanical origins of the pollen samples were confirmed by palynological analysis and DNA barcoding. Then, physicochemical, phytochemical, and advanced chromatographic analyses (LC–MS/MS) were performed to reveal the bioactive compound contents and nutrient profiles of the samples in detail. When the polyphenol profile was analyzed, the highest level of hyperoside (11,071.28 mg/kg) was detected in monofloral sunflower pollen and rutin (1287.16 mg/kg) in the polyfloral sample. The most dominant amino acid in both pollen types was proline, which was measured as 4652.08 mg/kg (monofloral) and 12,476.82 mg/kg (polyfloral), respectively. Moreover, both samples contained remarkable levels of folic acid (vitamin B9; 20.80 and 20.77 mg/kg). The results obtained from this study reveal that bee pollen derived from the widely cultivated sunflower plant also possesses remarkable potential for the development of functional foods and nutraceutical products. However, as the study was conducted solely with monofloral and polyfloral bee pollen samples collected from a single geographical region, it can be stated that the findings should be considered as part of a preliminary evaluation. Despite this limitation, the data obtained through comprehensive analytical methods provide a scientific basis for the functional applications of monofloral sunflower pollen with its biologically valuable components.

## Graphical abstract

This study characterized monofloral sunflower bee pollen using palynological, physicochemical, and metabolomic analyses. LC–MS/MS results revealed significantly higher hyperoside levels in the monofloral pollen. The data obtained support the potential of sunflower pollen in functional food formulations.

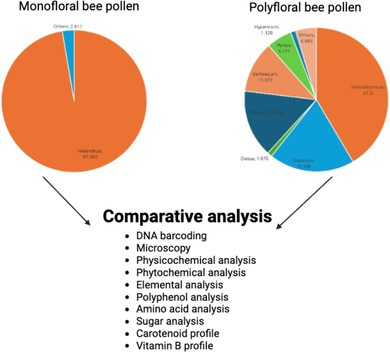

## 1 Introduction

Pollen is the male reproductive organ of flowering plants (Bayram et al. 2021). Flower pollen, a dust‐like substance, is collected by the honeybee *Apis mellifera* . The flower pollen is transferred into pollen baskets and placed in combs when it reaches the hive (Krell 1996). Since pollen is the primary food source for bees, its transportation to the hive is very important. When flower pollen is collected by bees, it is combined with some salivary enzymes secreted by bees, covered with honey in the hive, and transformed into bee pollen (Erdoğan and Dodoloğlu 2005). In addition to bees, pollen is also an important functional food for humans thanks to its high antioxidant content (Denisow and Denisow‐Pietrzyk 2016). Bee pollen has been found to be rich in bioactive substances such as carbohydrates, proteins, lipids, fatty acids, vitamins, minerals, and phenolic substances (Yang et al. 2013). The nutritional content of bee pollen may vary due to reasons such as plant origin and climatic conditions (AL‐Kahtani 2017). In addition, the storage conditions of pollen can also change the content of pollen (Bakkaloğlu 2021). Bee pollen can be monofloral or polyfloral depending on the plant source from which it is collected. In a previous study, it was determined that the content of monofloral bee pollen is more homogeneous compared to polyfloral pollen (Bogdanov 2012). However, since bees visit many different plant species during pollination, monofloral pollen is more difficult to access. To avoid this complexity, a previous study defined monofloral bee pollen as consisting of 80% or more of the same plant taxon (Campos et al. 2008). Bee pollen contains 5%–60% protein, 4%–7% lipid, 13%–55% carbohydrate, and 0.3%–20% fiber (Martinello and Mutinelli 2021). In addition, bee pollen contains vitamins such as β‐carotene, B1, B2, B3, B5, B6, B7, B9, vitamin C, and minerals such as Fe, Mg, Cu, Mn, K, C, P, Zn (Thakur and Nanda 2020). The chemical composition of bee pollen is composed of 1.6% phenolic compounds, 1.4% flavonoids and derivatives, and 0.2% phenolic acids (Komosinska‐Vassev et al. 2015). In addition, bee pollen has antimicrobial, anticancer, antifungal, and antioxidant properties thanks to this unique content (Fatrcová‐Šramková et al. 2013). It is very important to determine the bioactive substance profile of bee pollen, which is consumed as a functional food thanks to its rich phenolic substance, carotenoid, vitamin, mineral, amino acid, and fatty acid content. The nutrient content of bee pollen may vary due to reasons such as plant origin and climatic conditions. For this reason, it is important to determine the plant origin of pollen.

In this study, the botanical origins of monofloral sunflower and polyfloral bee pollen samples were confirmed by palynological and DNA barcoding methods, followed by physicochemical (moisture, protein, lipid, carbohydrate, ash, color, and energy content) analysis of the samples, phytochemical (total phenolics, flavonoids, proanthocyanidins, total carotenoid, and antioxidant capacity) and chromatographic (polyphenols, amino acids, vitamins, sugars, carotenoids, elements, and fatty acids) analyses were performed. In this context, the metabolomic profiles of monofloral sunflower pollen and polyfloral pollen samples collected from Turkey are presented in detail. In addition to determining the quality of pollen samples, the data obtained within the scope of the study also provide scientific data for determining their botanical and geographical origins. However, since this study was conducted on only one monofloral and one polyfloral bee pollen sample collected from a single geographical region, it is recommended that the findings obtained should be evaluated within the scope of a preliminary study within the limitation of the number of samples and regional coverage.

## 2 Material and Methods

### 2.1 Sample Preparation

One monofloral and one polyfloral pollen sample collected from the Thrace region of Turkey were stored at −20°C until the experiments were performed. Samples for phytochemical experiments were prepared using an ultrasonic extraction system as recommended by Zhou et al. (2015).

### 2.2 Palynological Analysis and DNA Barcoding

For botanical origin identification, images were first taken under a microscope and then DNA isolation was performed using the HiMeadi‐HiPurA Multiple Sample Purification Kit according to the manufacturer's instructions. In the PCR study for DNA barcoding, rbcLa‐F and rbcLa‐R primers were used to amplify the gene regions targeted for species identification. MAGIO “HighPrepTM PCR Clean‐up System” (AC‐60005) kit was used for purification of PCR products, and the procedure was performed as described in the kit. Metagenome analysis was performed by Sanger sequencing in the Macrogen Netherlands laboratory using an ABI 3730XL instrument (Applied Biosystems, Foster City, CA) and the BigDye Terminator v3.1 Cycle Sequencing kit.

### 2.3 Physicochemical Analyses

Moisture determination was carried out in accordance with the AOAC 920 181‐1920 method (AOAC 1997). A 1 g sample was completely dried for about 24 h using a lyophilizer. The dried samples were weighed, and the moisture content was calculated as a percentage. Protein quantification of the samples was performed using the Bradford method (Bradford 1976). Prior to Bradford analysis, homogenization of the samples was performed using the method proposed by Mæhre et al. (2018). Total soluble protein results were calculated as a percentage. The lipid contents of pollen samples were analyzed using the Bligh and Dyer method (Bligh and Dyer 1959). The results were presented as percentages. Total carbohydrate analysis was performed according to the method proposed by Yang et al. (2013). Total carbohydrate content was calculated as a percentage. The ash content of the samples was determined according to the AOAC 920181‐1920 method (AOAC 1997). The results obtained are presented as percentages. L* (lightness), a* (red/green), and b* (blue/yellow) values of bee pollen samples were determined using the NH300 colorimeter (3nh, Guangzhou, China) based on the CIELAB (Lab) color system. The energy calculation of bee pollen samples was carried out using the Atwater method described in Merrill and Watt (1955).

### 2.4 Phytochemical Analyses

The total phenolic content of pollen samples was determined using the Folin–Ciocâlteu microplate method (Magalhães et al. 2010). Total phenolic content results were expressed as gallic acid equivalents (mg GAE/g). Total flavonoid content was determined by the AlCl_3_ colorimetric method with modifications described by Kostić et al. (2021) and Chang et al. (2002). The results obtained were expressed in quercetin equivalence (mg QE/g). Total proanthocyanidin content was determined based on the method described by Broadhurst and Jones (1978), modified to a total assay volume of 500 μL. Total proanthocyanidin content was expressed as catechin equivalence (mg CE/g). In order to determine the total carotenoid content, bee pollen samples were extracted according to the method proposed by Gu et al. (2008). Total carotenoid content was measured according to the methods described by Kostić et al. (2021) and Fikselová et al. (2008). Results are expressed in β‐carotene equivalents (mg β‐CAR/g). In order to determine the total antioxidant capacity of bee pollen samples, CUPRAC, DPPH, ABTS, and CERAC tests were performed according to the methods proposed by Apak et al. (2004), Bobo‐García et al. (2015), Re et al. (1999), and Ozyurt et al. (2007), respectively. The results were expressed in terms of trolox equivalence (mg TE/g).

### 2.5 Chromatographic Analyses

#### 2.5.1 Polyphenol Analysis

##### 2.5.1.1 Extraction of Polyphenols

Polyphenol analysis of the samples was performed according to the method proposed by Zhou et al. (2015). The extracted samples were purified using Sep‐Pak C18 purification cartridges. The purified samples were homogenized using chromatography solvent (water:methanol:formic acid, 80:19:1, v/v/v) before loading into the LC–MS/MS system.

##### 2.5.1.2 LC–MS/MS Conditions

LC–MS/MS analyses were performed using a Thermo TSQ Quantis instrument (Thermo Scientific, Waltham, MA, USA) equipped with an electrospray ionization probe. Substance separation was performed with a Thermo Accucore C18 HPLC column (150 mm × 2.1 mm, 2.6 μm). Autosampler temperature was 4°C, column temperature was 30°C, flow rate was 0.2 mL/min, mobile phase A was 5 mM ammonium acetate, and mobile phase B was acetonitrile: methyl alcohol (1:1, v:v) solution consisting of 1% acetic acid. The injection volume was 5 μL, and the total analysis time was 28 min (Ecem Bayram 2021). The mobile phase flow was started with 5% B, and the gradient was adjusted from minute 2.1 to 100% B at minute 16.0. After reaching 100% B at 16.0 min, the B phase was kept constant until 20.0 min and reduced to 5% B at 20.01 min. The 5% B phase was kept constant until 28.0 min to maintain the column pressure. MS parameters were set so that the spray voltage was 3 kV in positive and negative mode, sheath gas flow was 54 arb, auxiliary gas flow was 11 arb, sweep gas was 1 arb, ion transfer tube temperature was 275°C, and evaporation temperature was 400°C. Data acquisition and processing were performed with Chromeleon 7.1 (Thermo LC–MS software; Gercek et al. 2024).

#### 2.5.2 Amino Acid Analysis

##### 2.5.2.1 Extraction of Amino Acids

1 mL of methanol:dH_2_O:formic acid (vv, 80:20:1) was added to 20 mg of bee pollen and then extracted with a magnalyzer (Roche, Basel, Switzerland) in two steps at 6000 rpm for a total extraction time of 50 s. Then, the supernatants of the samples centrifuged at 21,913 g for 30 min (Eppendorf 5417R centrifuge (Eppendorf, Hamburg, Germany), F45‐30‐11 rotor) were transferred to a new centrifuge tube. To the residue, 1 mL of extraction solvent was added and the magnalyzer process was repeated. The well‐disintegrated samples were centrifuged at 21,913 g for 30 min. After centrifugation, the supernatants were combined. The resulting extract was kept at −40°C for 1 h and then centrifuged at 11,180 g, 4°C for 20 min. The supernatants were diluted with chromatography solution (methanol:dH_2_O:formic acid, v:v, 80:20:1), transferred to vials, and loaded into LC–MS/MS for reading.

##### 2.5.2.2 LC–MS/MS Conditions

Amino acid analyses were performed in accordance with the methods previously described by Ecem Bayram et al. (2021) and Mayr and Schieberle (2012). LC–MS/MS analyses were performed using a Thermo TSQ Quantis instrument equipped with an ESI ion source. Chromatographic separation was performed using a Thermo Accucore HILIC HPLC column (150 mm × 3.0 mm, 2.6 μm particle size) at a flow rate of 0.6 mL/min and a column oven temperature of 35°C. As the mobile phase, 5 mM ammonium formate solution containing 2% formic acid (mobile phase A) and acetonitrile containing 0.1% formic acid (mobile phase B) were used. The gradient was maintained for 1 min starting with 80% phase B and decreased linearly to 25% in 4 min. This ratio was maintained for 1 min and returned to the initial ratio at 5.1 min. The total duration of the method was 10 min. The autosampler temperature was maintained at 4°C. The injection volume was set to 5 μL. Measurements of all amino acids were performed with a spray voltage of 4500 V with the polarity of the ion source in positive mode. MS parameters were set to sheath gas 45 arb, auxiliary gas 10 arb, ion transfer tube temperature 270°C, and evaporation temperature 300°C. Data acquisition and processing were performed using Chromeleon 7.1 (Thermo LC–MS software).

#### 2.5.3 Vitamin B Analyses

##### 2.5.3.1 Vitamin B Extraction

10 mL hexane was added to a 200 mg pollen sample and vortexed for 10 s. The vortexed pollen samples were kept in an ultrasonic water bath for 10 s. After the water bath, pollen samples were centrifuged at 10,304 g for 10 min, and the supernatants were discarded. The remaining pellets were dried at low temperatures. To the dried pellets, 5 mL of methanol containing 0.2% formic acid was added, and the sample tubes were placed on a shaker for 5 min. After incubation, the samples were extracted in an ultrasonic water bath for 10 min. The extracted samples were centrifuged at 10,304 g for 10 min, and the supernatants were transferred to balconies. The volume of the samples was completed to 10 mL. The pollen samples were then centrifuged again at 10,304 g for 10 min, and the supernatants were transferred to vials through 0.22 μm PTFE filters and loaded into the LC–MS/MS system.

##### 2.5.3.2 LC–MS/MS Conditions

Vitamin B assays were performed using a Thermo TSQ Quantis instrument equipped with an ESI probe under the conditions previously described by Çelik et al. (2024), with some modifications. A Thermo Accucore C8 column (150 mm × 2.1 mm, 2.6 μm particle size) was used for sample separations. Liquid chromatography (LC) conditions were set as column oven temperature 40°C, flow rate 0.4 mL/min and injection volume 5 μL. Mobile phases were water containing 5 mM ammonium formate and 0.1% formic acid (mobile phase A) and methanol containing 0.1% formic acid (mobile phase B). The flow gradient of the method was 5% B at 0 min, 5% B at 0.7 min, 95% B at 2.0 min, 95% B at 6.5 min, 5% B at 6.60 min, and 5% B at 11.00 min. The total method time was 11 min. Mass spectrometry (MS) conditions for the analyses were set as follows: positive ion voltage 3000 V, negative ion voltage 3500 V, sheath gas flow 54 arb, auxiliary gas flow 15 arb, sweep gas flow 1 arb, ion transfer tube temperature 275°C and evaporation temperature 375°C. Vitamin B analyses were performed using Chromeleon 7.1 software.

#### 2.5.4 Carotenoid Analysis

##### 2.5.4.1 Extraction of Carotenoids

To 50 mg of homogeneous bee pollen samples, 1 mL of extraction solution (hexane:acetone, 1:1, v:v) was added, and after vortexing for 2 min, the mixture was centrifuged at 11,180 g for 10 min. The supernatant was transferred to a known‐dimensional vial, and this process was repeated until a colorless extract was obtained. The extraction solvent was removed using evaporation under nitrogen, keeping the system temperature below 30°C. KOH (20%, v/v) dissolved in 500 μL dichloromethane and 500 μL methanol was added to the solvent‐removed carotenoid extract and incubated at room temperature for 1 h in the dark. Saponified extracts were washed with 5% NaCl (w/v) until a neutral pH was reached. Dichloromethane was evaporated under nitrogen, and the water phase was removed from the extract using a lyophilizer. Carotenoids were dissolved with 400 μL ethyl acetate and transferred to vials.

##### 2.5.4.2 LC–MS/MS Conditions

Chromatographic analysis of carotenoids was performed using a Thermo TSQ Quantis LC–MS/MS instrument equipped with an ESI probe. The chromatographic separation of the substances was completed with a Thermo Accucore C18 (150 mm × 2.1 mm, 2.6 μm) column. The column oven temperature was set at 35°C, and the injection volume was 10 μL. Methanol containing 0.02% formic acid (Mobile phase A) and acetonitrile (Mobile phase B) were introduced into the system with isocratic flow at a mobile phase ratio of 5B%. The total method time was set as 5 min. MS parameters were set as follows: positive ion voltage 4000 V, sheath gas flow 30 arb, auxiliary gas flow 5 arb, sweep gas flow 1 arb, ion transfer tube temperature 150°C, and evaporation temperature 350°C (Zhang et al. 2019). Data were obtained and organized using Chromeleon 7.1 software, and results are expressed in mg/kg.

#### 2.5.5 Sugar Analysis

##### 2.5.5.1 Extraction of Sugars

After adding 10 mL of distilled water to 500 mg of pollen sample, the mixture was kept in an ultrasonic water bath for 30 min. 2 mL of this mixture was transferred to a centrifuge tube and 1.5 mL of distilled water was added. After incubating the mixture at 60°C for 10 min, 0.25 mL of Carrez I and Carrez II solutions and 1 mL of acetonitrile were added, and the tubes were shaken gently and left at room temperature for 1 h without shaking. After the waiting period, the tubes were centrifuged at 10,000 g for 8 min at 20°C. The resulting supernatant was transferred to vials through a 0.45 μm nylon membrane filter (Sharma et al. 2009).

##### 2.5.5.2 HPLC‐RID Conditions

Chromatographic analysis of sugars was performed using the HPLC‐RID system. Chromatographic separation of sugars was performed on a Thermo Hypersil GOLD Amino column (150 mm length, 4.6 mm diameter, 3 μm particle size) with an injection volume of 10 μL and a mobile phase flow of 1.2 mL/min. Acetonitrile: ultrapure water (80:20 v/v) was used as the mobile phase, and the column oven temperature was set to 45°C. The detector temperature was 35°C, and detector scanning was performed with data acquisition parameters of 1 s and 5 Hz. Data were acquired and organized using Chromeleon 7.1 software, and results were expressed in mg/kg (Gercek et al. 2024).

#### 2.5.6 Elemental Analysis

##### 2.5.6.1 Digestion Procedure

The lyophilized 300 mg samples were transferred to Teflon containers and 5 mL of 65% nitric acid was added. After shaking, the samples were kept at room temperature for 20 min. Incineration was performed in three stages using the Berghof Microwave System. In the first stage, the temperature was 145°C, the power was 75%, and the duration was 5 min. In the second stage, the temperature was set to 190°C, power 90%, and time 10 min. In the third stage, the temperature was set at 100°C, the power was 40%, and the duration was 10 min. In this way, the incineration process was completed in 25 min in total. After the incineration procedure was completed, the Teflon containers were allowed to cool down to room temperature. The samples were then transferred through 0.45 μm nylon filters into 50 mL balloon jugs, and the total volume was made up to 50 mL with 65% nitric acid. Samples were stored at +4°C until analysis.

##### 2.5.6.2 ICP‐OES Conditions

Calibration solutions were prepared using single element certified reference materials (CRMs) and ICP‐multielement Standard Solution VIII (Supelco) with elemental concentrations between 5 μg/kg and 50 mg/kg. A blind solution was used to determine the elemental composition of the samples, and the samples were compared with this blind solution. All sample readings were performed in triplicate. The presence of the elements B, Ba, Be, Bi, Ca, Co, Cr, Cu, Fe, K, Li, Mg, Mn, Na, Ni, P, Se, Zn in the sample was determined using a Perkin Elmer Optima 7000DV model ICP‐OES (Waltham, MA, USA) operating at 27.12 MHz, adjusted between 700 W and 1400 W in 100 W increments under constant values of RF power and other plasma conditions. Peristaltic pump values were set at 50 rpm for both analysis and washing; nebulizer gas flow at 0.7 L/min; coolant gas flow at 12 L/min; auxiliary gas flow at 0.5 L/min; plasma mode in dual (vertical/horizontal) view; and sample acquisition delay at 30 s. The nebulizer argon flow rate was increased from 0.5 to 1.5 mL/min in 0.5 mL/min intervals. Both axial and radial imaging modes were considered as they can affect the magnitude of matrix effects and the signal‐to‐background ratio.

#### 2.5.7 Fatty Acid Analyses

##### 2.5.7.1 Methyl Esterification of Fatty Acids

Lipid samples obtained by the method described by Bligh and Dyer (1959) were dissolved 1:100 with n‐hexane solvent and transferred to centrifuge tubes. Then, 2 N KOH dissolved in 100 μL methanol was added to the samples and vortexed for 2 min. After vortexing, the samples were centrifuged at 412 g for 10 min and 1 mL of the supernatant was separated for GC–MS analysis (David et al. 2002).

##### 2.5.7.2 GC–MS Conditions

Methyl esterified fatty acids were analyzed using an Agilent 7890A GC gas chromatograph and 5975C MSD mass spectrophotometer. HP‐88 (100 m × 0.25 mm ID, 0.25 μm) column and helium as carrier gas were used for separation. For the chromatographic separation of fatty acids, 1 μL of sample was injected with a 1/20 split ratio, and the column oven temperature was held at 120°C for 1 min and then increased to 175°C within 10 min. The column was kept at this temperature for 10 min, then the oven temperature was increased to 210°C in 50°C increments over 5 min and kept at 210°C for 5 min. The oven temperature was then raised to 240°C in 5°C increments over 3 min (Regulation 1991). In this way, the chromatographic separation of fatty acids in gas chromatography was completed. MS analyses were performed at a detector temperature of 280°C and a detector gas flow of 30 mL/min. Supelco 37 Component FAME Mix (Sigma Code CRM47885) was used as a standard. Sample peaks were identified according to the retention time of the substances in the standard (Bastürk et al. 2024).

### 2.6 Statistical Analysis

Statistical analysis was conducted using a two‐sample *t*‐test (GraphPad Prism 10.1.1, USA) to assess significant differences between the samples. All tests were performed in triplicate, and the results are presented as mean ± SD.

## 3 Results and Discussion

### 3.1 Botanical Origin

**FIGURE 1:**
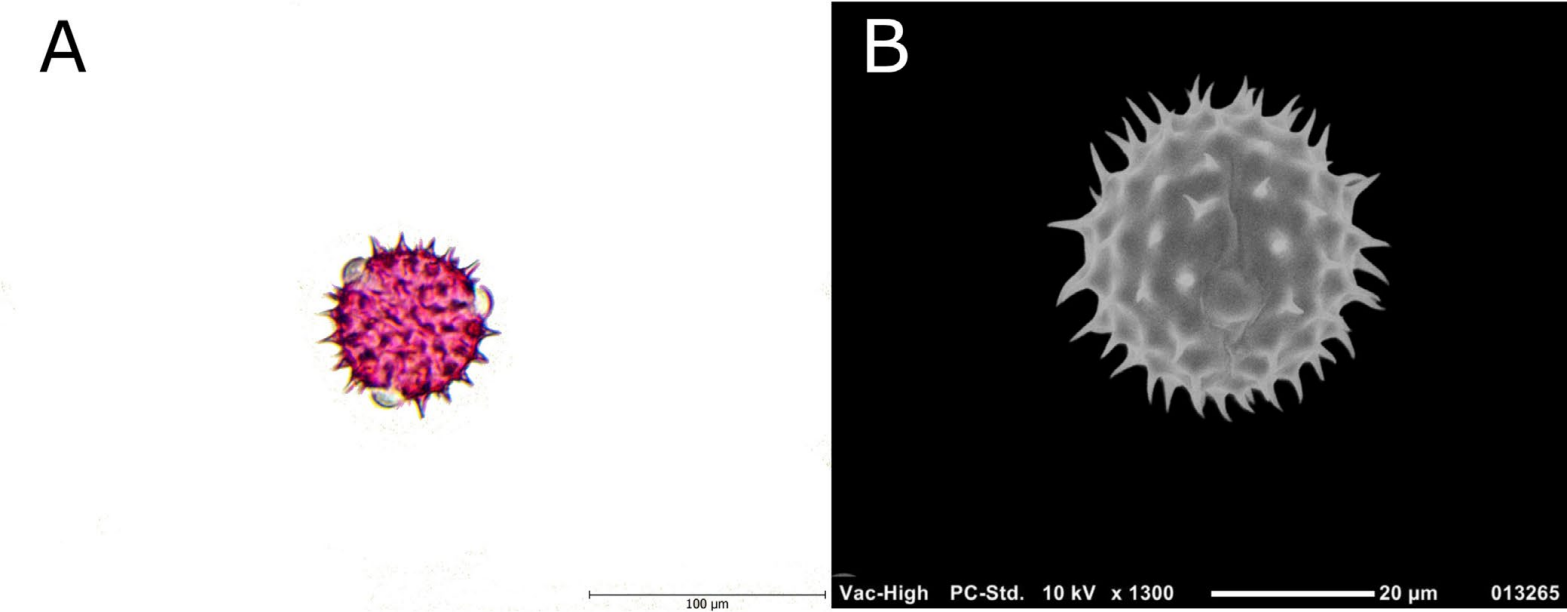
(A) Light microscopy image of *Helianthus annus* pollen. (B) SEM image of *Helianthus annus* pollen.

**FIGURE 2:**
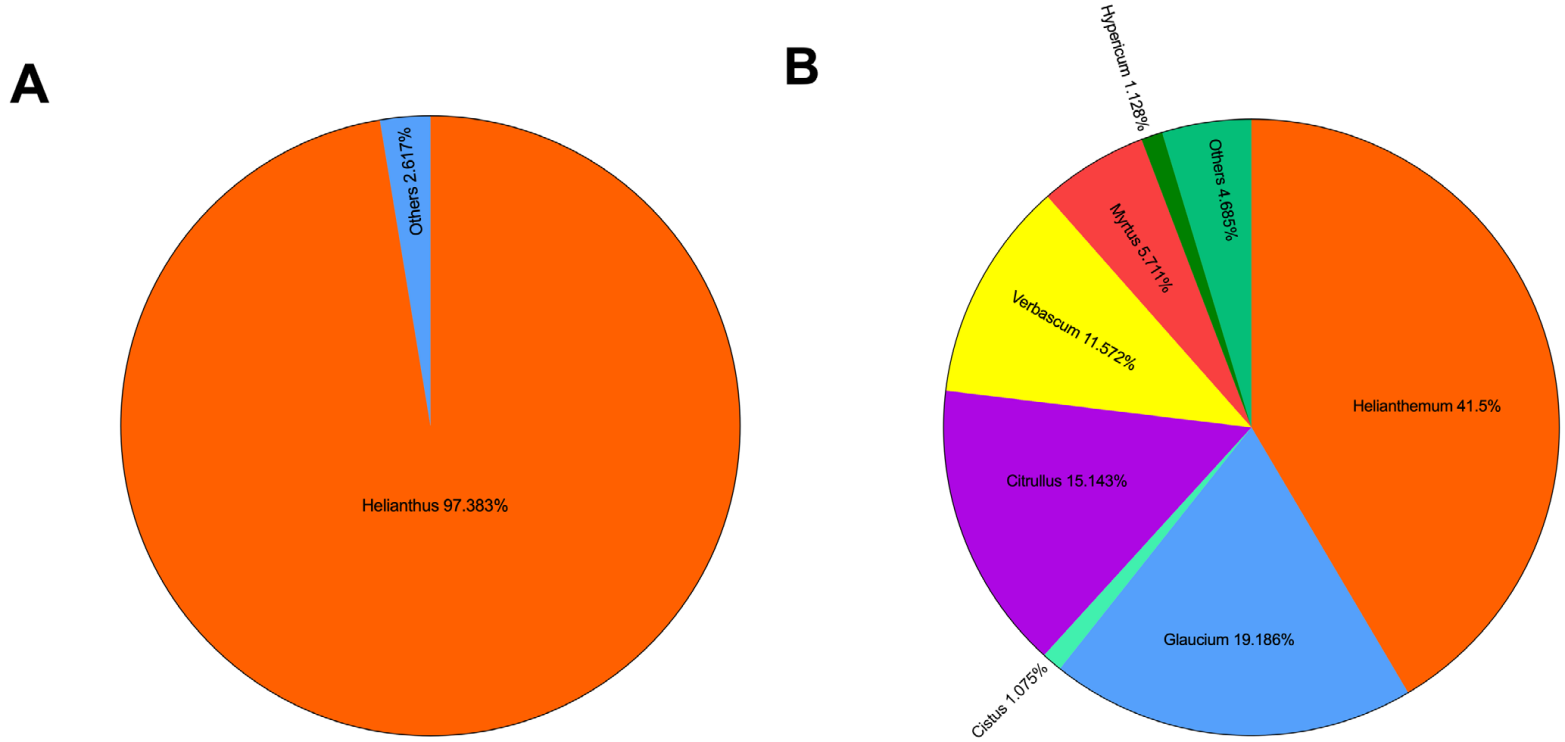
Percentages of plant genus in monofloral (A) and polyfloral pollen (B) according to DNA barcoding results.

Within the scope of the study, palynological analyses and molecular DNA barcoding methods were applied in an integrated manner to confirm the botanical origin of bee pollen samples. As a result of palynological examinations, it was determined that one of the two different pollen samples examined was monofloral sunflower ( *Helianthus annuus* ) pollen (Figure 1A,B) and the other was a pollen mixture with polyfloral character. DNA barcoding analysis revealed that the monofloral sample contained 97.38% *Helianthus* spp., 1.68% *Forsythia* spp., 0.1% *Salix* spp., and 0.84% other plant pollen (Figure 2A). Differently, 1.68% *Forsythia* spp. pollen detected by barcoding was not observed in palynological analysis. This may be due to the limitations of palynological analysis in making taxonomic distinctions. Campos et al. (2008) stated that for a pollen sample to be classified as monofloral, it should contain at least 80% pollen of the same plant genus. In line with this criterion, the sample containing 97.38% *Helianthus* spp. pollen in our study was definitely defined as monofloral. On the other hand, DNA barcoding analysis of the polyfloral pollen sample showed that the sample contained pollen from different plant taxa. These taxa included *Helianthemum* spp. (41.5%), *Glaucium* spp. (19.19%), *Citrullus* spp. (15.14%), *Verbascum* spp. (11.57%), *Myrtus* spp. (5.71%), *Hypericum* spp. (1.13%), *Cistus* spp. (1.08%), and other species (4.69%) (Figure 2B). The absence of any dominant plant species in these results for the polyfloral pollen sample (at least 80% belonging to the same plant genus) confirmed the classification of the sample as polyfloral.

### 3.2 Physicochemical Characterization

Two samples, monofloral sunflower bee pollen and polyfloral bee pollen, were used in the experiments. The results of the physicochemical analysis of bee pollen samples are summarized in Table 1. In this context, total soluble protein, lipid, and carbohydrate contents were analyzed to determine the nutritional values of the samples. Each sample was analyzed in triplicate.

**TABLE 1:** Physicochemical analysis of bee pollen samples.

	Monofloral sunflower^a^	Polyfloral^a^
Protein (%)	5.734 ± 0.227	5.725 ± 0.135
Lipid (%)	5.54 ± 0.341	5.583 ± 0.242
Carbohydrate (%)	87.271 ± 0.076	86.199 ± 0.67
Ash (%)	1.456 ± 0.116	2.054 ± 0.073
Moisture (%)	7.138 ± 0.114	0.844 ± 0.084

^a^ Mean ± SD of triplicate values.

Bee pollen, which is rich in protein, is an important component for both honey bees and human nutrition. In general, bee pollen has a protein content ranging from 12% to 30%. Pollen with a crude protein content above 25% is classified as high quality, those between 20% and 25% as medium quality, and those below 20% as low quality (Bayram et al. 2024). In this study, it was found that the soluble protein content of monofloral bee pollen was 5.73% ± 0.23% and the soluble protein content of polyfloral bee pollen was 5.73% ± 0.13%. These results indicate that both samples belong to the low‐quality pollen group. Nicolson and Human (2013) also reported that sunflower pollen has a low protein content and is therefore considered a low‐quality pollen. Similarly, Mayda et al. (2020) reported that the protein content of pollen samples of different plant origin varied between 17.6% and 22.2%. However, in a study investigating bee pollen samples obtained from different origins, it was reported that the crude protein content of pollen samples varied between 18.62% and 26.51%, and the highest protein content was found in chestnut pollen (Kolayli et al. 2024). These differences in protein content are attributed not only to plant origin and environmental factors but also to various factors such as harvest time and processing methods (Bobiş et al. 2025; Boulfous et al. 2025).

Bee pollen is a matrix rich in carbohydrates and lipids as well as proteins. Approximately two‐thirds of pollen is composed of carbohydrates. In this study, the carbohydrate content of monofloral sunflower pollen and polyfloral pollen was 87.27% ± 0.076% and 86.2% ± 0.67%, respectively. Lipid contents were 5.54% ± 0.34% and 5.58% ± 0.24%, respectively. Our findings are in line with the reports of Campos et al. (2008) and reveal that the carbohydrate content varies between 13% and 55% and lipid content between 1% and 13% depending on the botanical and geographical origin of the pollen. Similarly, it has been reported that the total lipid content of bee pollen samples collected from different seasons and apiaries ranged between 4.37% and 13.17%, and that monofloral *Brassica* spp. pollen had a higher lipid content than other samples (Gámbaro et al. 2025). This indicates that, in addition to botanical origin, the lipid content of pollen also varies significantly depending on harvest time (Gámbaro et al. 2025).

Moisture content is an important parameter affecting the susceptibility of pollen to microbial contamination. In this study, 7.14% ± 0.11% moisture was detected in monofloral bee pollen and 0.84% ± 0.084% in polyfloral pollen. Thakur and Nanda (2020) reported that the moisture content of bee pollen varies between 7% and 30%. In a study conducted on pollen obtained from Turkey, the moisture content was found to be between 8% and 28% (Isik et al. 2018). In *Helianthus annuus* pollen obtained from Romania, 26.36% moisture content was reported (Spulber et al. 2018). In the same study, the moisture content of 10 pollen samples of different plant origin varied between 16.92% and 31.08%. Similarly, the moisture content of bee pollen samples with different botanical and geographical origins was determined to be between 6.71 and 16.67 (Mutlu et al. 2025).

In the study, ash content was also determined; it was measured as 1.46% ± 0.12% in monofloral pollen and 2.05% ± 0.07% in polyfloral pollen. Campos et al. (2008) reported that the ash content of pollen can be between 2% and 6%, while Kaur et al. (2024) reported that it varies between 3.3% and 4.4%. Additionally, it has been determined that the ash content of different monofloral and multifloral pollen samples varies. Accordingly, in a related study, the ash content of mustard pollen was found to be 4.3%, while that of multifloral pollen was determined to be 3.3% (Kaur et al. 2024).

Bee pollen has the potential to support daily energy needs thanks to its high nutrient content. Although the daily energy requirement varies according to the age, gender, and physical activity level of the individual, it is around 1600 kcal for children, 2100 kcal for women, and 2700 kcal for men (Karadoğan and Özer 1997). In this study, it was determined that the energy content of polyfloral pollen was higher than that of monofloral pollen.

Color analysis was performed in CIELab color space. L* value (luminance) was 59.52 and 58.25 for monofloral and polyfloral pollen, respectively. a* (green‐red) and b* (blue‐yellow) values were 16.59 and 28.82 for monofloral and 13.71 and 28.22 for polyfloral pollen, respectively. These results indicate that both pollen samples have similar color characteristics (Table 2).

**TABLE 2:** Color analysis results of bee pollen samples.

	Monofloral sunflower^a^	Polyfloral^a^
L*	59.52 ± 0.753	58.25 ± 0.71
a*	16.59 ± 0.915	13.71 ± 0.98
b*	28.82 ± 2.77	28.223 ± 1.92
C*	33.273 ± 2.572	31.40 ± 1.82
H*	59.943 ± 2.43	64.02 ± 2.091
Δ*E*	49.443 ± 1.25	49.126 ± 1.626

Abbreviations: ∆*E*, the difference between two colors in the Lab* space; a*, green‐red; b*, blue‐yellow; C*, chroma; H*, hue angle; L*, lightness.

^a^ Mean ± SD of triplicate values.

### 3.3 Phytochemical Content

**FIGURE 3:**
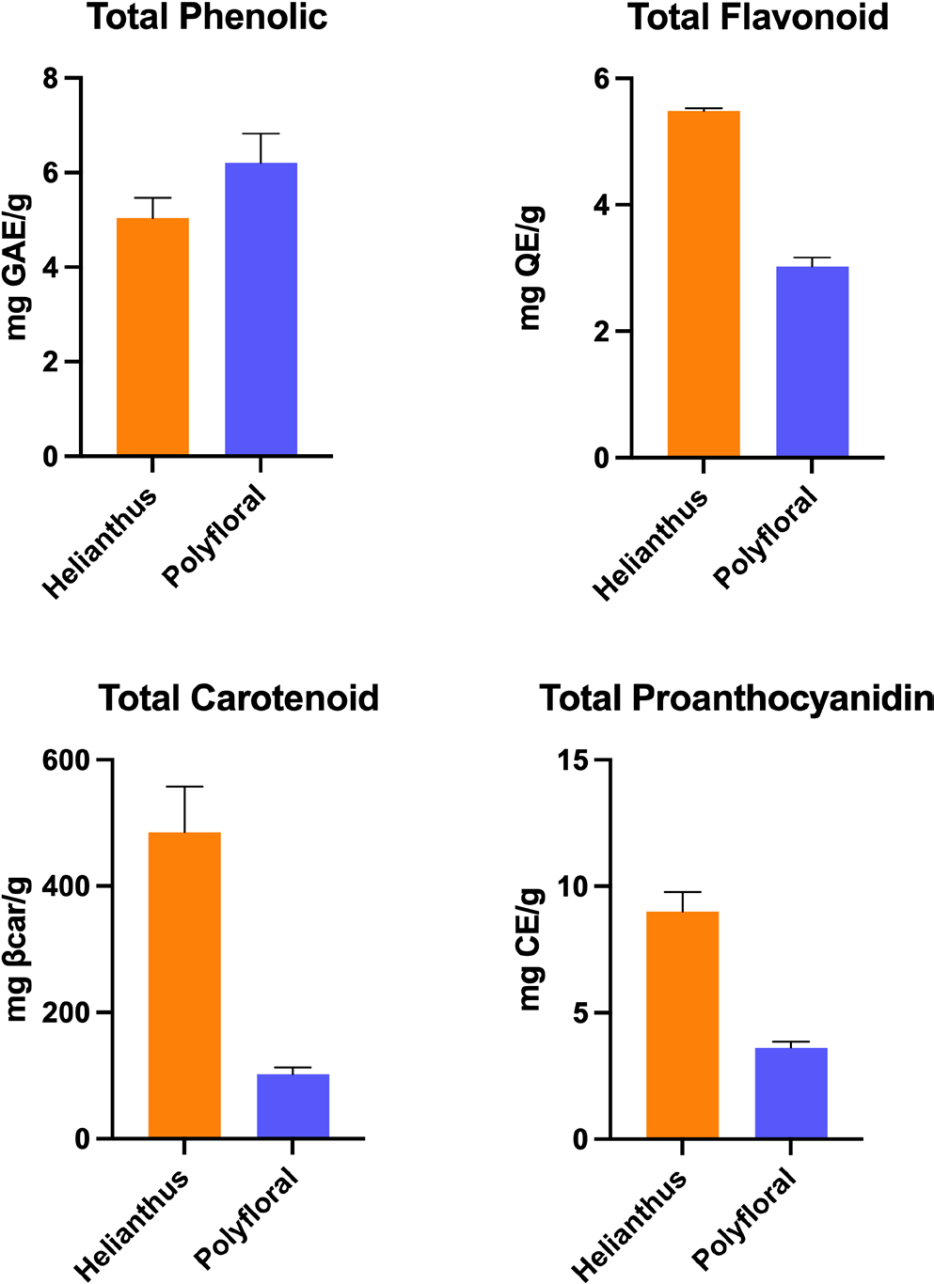
Phytochemical analysis results of monofloral and polyfloral bee pollen.

Although their deficiency does not lead to plant death, plant secondary metabolites play a role in physiological processes such as maintenance of vitality, reproductive success, growth and development, as well as numerous ecological functions such as defense against herbivores, pollinator attraction, and protection against UV radiation (Crozier et al. 2006; Tiwari and Rana 2015). Polyphenols, which constitute an important group of these compounds, are substances commonly found in plant metabolism, containing at least one aromatic ring and one or more hydroxyl groups. In the literature, more than 8000 phenolic compounds have been identified in plants (Strack 1997). Polyphenolic compounds have attracted great attention in recent years due to their natural antioxidant properties and their positive effects on health. Bee pollen stands out as a valuable natural product in this context because it contains phenolic acids and flavonoids. In this study, total phenolic content was determined as 5.03 mg GAE/g for monofloral sunflower bee pollen and 6.21 mg GAE/g for polyfloral bee pollen (Figure 3).

Flavonoids have roles in plants such as the coloration of flowers, formation of aromatic structures, and attraction of pollinators, as well as protection against biotic and abiotic stress factors, absorption of UV rays, and antimicrobial and antioxidant effects (Panche et al. 2016). In our study, total flavonoid content was determined as 5.486 mg QE/g for monofloral sunflower pollen and 3.027 mg QE/g for polyfloral pollen (Figure 3). In a bee pollen sample collected from Morocco where *Helianthemum* species were dominant, the amount of flavonoids was reported as 3.2 mg QE/g; it was also reported to be high in phenolic acids (Aylanc et al. 2023). Kaur et al. (2024) reported the total flavonoid content in pollens of different botanical origins (mustard and multifloral) as ranging from 14.025 mg QE/g for mustard pollen to 8.38 mg QE/g for multifloral pollen. These differences can be attributed to the botanical and geographical origin of the samples.

**FIGURE 4:**
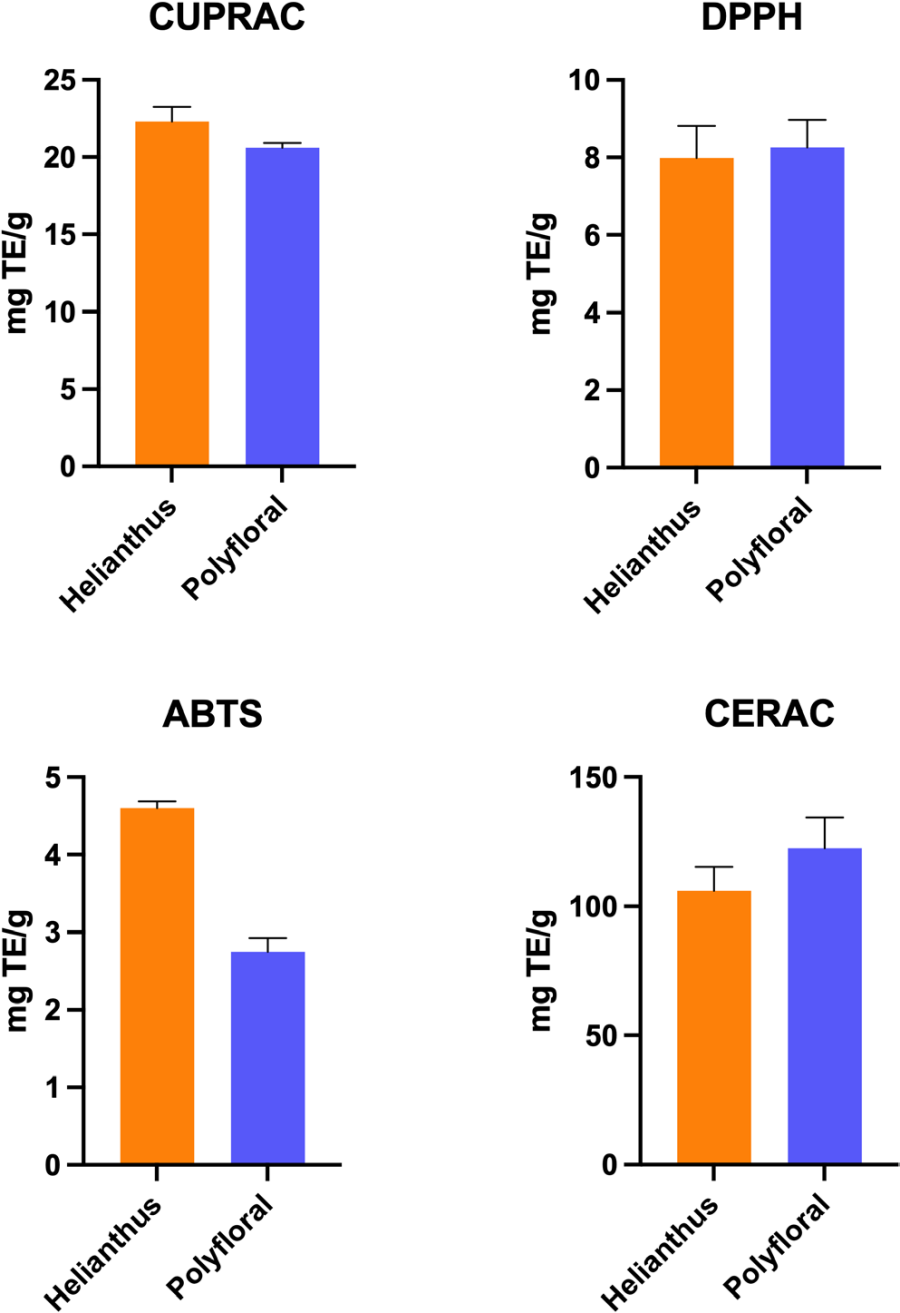
Total antioxidant content results of monofloral and polyfloral bee pollen.

CUPRAC, ABTS, DPPH, and CERAC methods were used to determine antioxidant capacity. In general, there was no significant difference between monofloral sunflower pollen and polyfloral samples, but in ABTS analysis, monofloral sunflower pollen (4.601 mg TE/g) had higher antioxidant capacity compared to polyfloral pollen (2.747 mg TE/g), and this difference was statistically significant (Figure 4). Fatrcová‐Šramková et al. (2013) also reported that sunflower pollen showed lower DPPH inhibition compared with other pollen types. The values we obtained in the CUPRAC analysis (monofloral: 22.299 mg TE/g; polyfloral: 20.621 mg TE/g) are compatible with the range of 8.8–22.98 mg TE/g reported by Ulusoy and Kolayli (2014).

Proanthocyanidins are condensed tannins found in parts of plants such as flowers, fruits, seeds, and bark that provide defense against biotic/abiotic stress. These compounds show antioxidant (Qian et al. 2025), antimicrobial (Mattos et al. 2024), anticancer (Li et al. 2024), and antidiabetic (Zeng et al. 2024) effects. In our study, proanthocyanidin content was measured as 9.01 mg CE/g in monofloral pollen and 3.24 mg CE/g in polyfloral pollen (Figure 3). These values are considerably higher than the value of 0.86 mg CE/g reported in pollen samples collected from Giresun (Kemal and Şahin 2020).

The yellow to red color of bee pollen is mostly due to pigments such as flavonoids and carotenoids (Stanley and Linskens 2012). Carotenoids are pigment molecules with antioxidant properties, and especially β‐carotene, lutein, and β‐cryptoxanthin are the most common ones in bee pollen (Mărgăoan et al. 2014). In this study, total carotenoid content was determined as 1316.334 μg β‐carotene/g for monofloral pollen and 255.289 μg β‐carotene/g for polyfloral pollen (Figure 3). Fatrcová‐Šramková et al. (2016) reported an average carotenoid content of 240 mg/kg in sunflower pollen under different storage conditions; this value is approximately one fifth of the value obtained in our study. Gardana et al. (2018) reported zeaxanthin contents of 24.7, 56.9, and 221.47 μg/g in bee pollen samples collected from Italy, Spain, and Colombia, respectively. The fact that the carotenoid levels obtained in our study were higher than these literature values indicates that carotenoid content may vary significantly depending on plant origin.

### 3.4 Polyphenolic Profile

Within the scope of the study, a total of 26 polyphenolic compounds in different bee pollen samples were analyzed qualitatively and quantitatively by the LC–MS/MS method (Table 3). The highest amount of phenolic compound detected in monofloral sunflower bee pollen was hyperoside with a value of 11,071.28 mg/kg. In polyfloral bee pollen, the polyphenol detected at the highest level was rutin with 1287.16 mg/kg.

**TABLE 3:** Polyphenol profile of bee pollen samples (mg/kg).

Polyphenols	Monofloral sunflower^a^	Polyfloral^a^
(−)‐Epicatechin	1.12 ± 0.1	0.44 ± 0.01
Apigenin	1.62 ± 0.03	172.37 ± 4.57
Artepillin C	0.07 ± 0.01	0.07 ± 0.01
Caffeic acid phenethyl ester	15.71 ± 0.09	1.68 ± 0.09
Catechol	1.2 ± 0.04	2.01 ± 0.14*
Chlorogenic acid	2.45 ± 0.22	3.72 ± 0.03*
Chrysin	21.32 ± 0.16	6.15 ± 0.31
Galangin	7.65 ± 0.2	1.21 ± 0.02
Gallic acid	0.7 ± 0.01	7.05 ± 0.09
Hesperidin	2.38 ± 0.18	23.55 ± 0.69
Hyperoside	11,071.28 ± 278.66	851 ± 18.19
Kaemferol	16.97 ± 0.81	420.13 ± 17.8
Luteolin	0.41 ± 0.02	1158.09 ± 63.79
Myricetin	9.21 ± 0.49	402.52 ± 8.51
Naringenin	2.35 ± 0.05	66.24 ± 1
Neochlorogenic acid	0.98 ± 0.07	1.58 ± 0.02
Pinobanksin	9.97 ± 0.12	19.87 ± 0.24
Pinocembrin	24.54 ± 0.28	3.52 ± 0.25
Protocatechuic acid	6.81 ± 0.67	10.93 ± 0.7*
Quercetin	208.18 ± 3.29	855.1 ± 12.93
Quercitrin	7.09 ± 0.22	852.07 ± 9.96
Rutin	84.38 ± 4.54	1287.16 ± 30.81
p‐coumaric acid	ND	0.24 ± 0.03
t‐cinnamic acid	1.9 ± 0.07	6.16 ± 0.12
trans‐caffeic acid	ND	ND
trans‐ferulic acid	3.83 ± 0.09	3.61 ± 0.07
Total	11502.12	6156.47

Abbreviation: ND, not detected.

^a^ Mean ± SD of triplicate values.

* *p* < 0.05.

Caffeic acid phenethyl ester (15.71 mg/kg), chrysin (21.32 mg/kg), galangin (7.65 mg/kg), hyperoside (11071.28 mg/kg), and pinocembrin (24.54 mg/kg) were found at statistically significantly higher concentrations in the monofloral sunflower pollen sample. In contrast, epicatechin (0.44 mg/kg), apigenin (172.37 mg/kg), protocatechuic acid (10.93 mg/kg), chlorogenic acid (3.72 mg/kg), gallic acid (7.05 mg/kg), hesperidin (23.55 mg/kg), kaempferol (420.13 mg/kg), myricetin (402.52 mg/kg), naringenin (66.24 mg/kg), neochlorogenic acid (1.58 mg/kg), pinobanksin (19.87 mg/kg), quercetin (855.1 mg/kg), quercitrin (852.07 mg/kg), rutin (1287.16 mg/kg), and t‐cinnamic acid (6.16 mg/kg), and these differences were considered statistically significant.

According to LC–MS/MS results, the total individual polyphenolic compound content of sunflower monofloral bee pollen was calculated as 11,502.13 mg/kg, while this amount was determined as 6156.46 mg/kg in polyfloral bee pollen. There are data compatible with these findings in the literature. For example, in a study on bee pollen from *Helianthus annuus* in Serbia, a total of 37 polyphenolic compounds were identified in ethanolic and methanolic extracts; 112.86 mg/kg of hyperoside was detected in the methanolic extract and 128.64 mg/kg in the ethanolic extract. As a result of this study, hyperoside was reported as the phenolic compound with the highest concentration (Kostić et al. 2019). The findings of Kostić et al. (2019) overlap with the data in our study; hyperoside was found to be the most dominant phenolic compound in both studies. However, the high amount of hyperoside detected in our study (11,071.28 mg/kg) can be explained by many factors such as the monoflorality rate of the sunflower pollen samples used, geographical origin, extraction parameters, processing conditions, and storage conditions. In another study conducted with different monofloral pollen samples collected from Turkey ( *Helianthus annuus* , *Cistus creticus* , *Papaver somniferum* , *Salix* sp.), the content of extracts prepared with various solvents was investigated in terms of 29 polyphenolic compounds. In this study, it was reported that the total amount of individual polyphenolic compounds in extracts of *Helianthus annuus* pollen ranged from 47 to 3213.3 mg/kg (Tel‐Çayan et al. 2025). The researchers detected the presence of the kaempferol compound (30.5–221.8 mg/kg) in all solvents used and stated that the presence and amount of other phenolic compounds differed depending on the solvent (Tel‐Çayan et al. 2025). The finding that the highest total individual phenolic content was obtained in the ethyl acetate fraction (Tel‐Çayan et al. 2025) demonstrates that compound profiles may vary depending on the type of solvent, and this highlights the importance of solvent‐specific compound extraction.

### 3.5 Amino Acid Profile

Amino acids are the basic building blocks that determine the chemical, physical, and biological properties of proteins (Whitford 2013). Amino acids such as phenylalanine, valine, threonine, tryptophan, methionine, isoleucine, leucine, lysine, and histidine are classified as essential and are considered as indispensable components for living life. Since these essential amino acids cannot be synthesized by the human body, they must be taken from outside through nutrition (Prandi et al. 2019).

According to the data reported by the World Health Organization (WHO), the amounts of essential amino acids that a healthy adult should take daily are 10 mg/kg for histidine, 20 mg/kg for isoleucine, 39 mg/kg for leucine, 30 mg/kg for lysine, 15 mg/kg for methionine + cysteine, 25 mg/kg for phenylalanine + tyrosine, 15 mg/kg for threonine, 4 mg/kg for tryptophan, and 26 mg/kg for valine (WHO 2007). Bee pollen stands out as a rich natural resource in terms of containing essential amino acids needed in human nutrition as well as components necessary for plant growth and development (Almeida‐Muradian et al. 2005).

In this study, 36 different individual amino acids were determined both qualitatively and quantitatively in the amino acid analysis of pollen samples (Table 4). Proline was found to be the amino acid with the highest level in both pollen types. In this context, proline content was 4652.08 mg/kg for monofloral sunflower bee pollen and 12,476.82 mg/kg for polyfloral bee pollen. It has been frequently reported in the literature that amino acid contents may vary depending on plant origin, geographical source, and seasonal variations (Yang et al. 2013; Taha et al. 2019; Al‐Kahtani et al. 2020; Alshallash et al. 2023). Within the scope of the study, the total essential amino acid content of sunflower pollen was calculated as 1.78 and 3.01 mg/kg for polyfloral pollen. These results indicate that the polyfloral pollen sample is richer in essential amino acids. In a study conducted in China and using bee pollen samples classified according to their plant origin, it was reported that the highest amino acids found in sunflower bee pollen were aspartic acid (11.3 mg/kg), glutamic acid (11.1 mg/kg), and proline (9.5 mg/kg), respectively (Yang et al. 2013). Similarly, the most dominant amino acids in sunflower bee pollen used in this study were proline (4652.08 mg/kg), glutamic acid (2768.74 mg/kg), aspartic acid (1891.18 mg/kg), and asparagine (1435.82 mg/kg). In a previous study, Bayram et al. (2021) examined the amino acid profiles of bee pollen samples collected from five different regions using LC–MS/MS. They determined the total FAA content to be between 48.8 and 64.2 mg/g.

**TABLE 4:** Amino acid profile of bee pollen samples (mg/kg).

Amino acid	Monofloral sunflower^a^	Polyfloral^a^
1‐Methylhistidine	310.59 ± 4.78	78.98 ± 1.38
3‐Methylhistidine	1.82 ± 0.14	ND
Anserine	ND	ND
Arginine	26.75 ± 0.22	16.21 ± 0.26
Asparagine	1435.82 ± 2.83	4010.43 ± 87.18
Aspartic acid	1891.18 ± 71.17	4462.64 ± 108.58
Carnosine	1.03 ± 0.02	0.15 ± 0.01
Citrulline	0.8 ± 0.02	16.52 ± 0.48
Cystathionine	0.16 ± 0.02	ND
Cystine	2.13 ± 0.21	5.78 ± 0.49
Etanolamine	777.43 ± 22.38	324.7 ± 5.94
Glutamic acid	2768.74 ± 102.13	2874.56 ± 95.01
Histidine	ND	ND
Homocystine	494.43 ± 11.63	177.64 ± 6.74
Hydroxylysine	159.84 ± 0.06	160.1 ± 0.12*
Hydroxyproline	148.96 ± 6.86	227.64 ± 7.15
Isoleucine	164.15 ± 6.76	271.52 ± 9.72
Lysine and allo‐isoleucine	125.89 ± 2.1	198.76 ± 6.01
Lysine	128.84 ± 1.59	184.04 ± 4.59
Methionine	201.39 ± 3.51	134.76 ± 0.43
Ornithine	104.87 ± 0.47	ND
Phenylalanine	301.65 ± 7.22	371.37 ± 6.67
Phosphoethanolamine	11.86 ± 1.13	37.26 ± 1.44
Phosphoserine	1.68 ± 0.12	ND
Proline	4652.08 ± 92.55	12,476.82 ± 302.51
Serine	669.06 ± 30.19	1010.2 ± 36.31
Taurine	129.77 ± 4.12	523.93 ± 13.08
Treonine	295.7 ± 9.5	488.19 ± 16.69
Tryptophan	44.71 ± 2.52	457.54 ± 15.48
Tyrosine	123.6 ± 5.79	220.34 ± 9.12
Valine	397.43 ± 13.69	681.7 ± 14.89
alpha‐aminoadipic acid	70.94 ± 4.45	34.39 ± 1.25
alpha‐aminobutyric acid	9.23 ± 0.65*	8.24 ± 0.45
beta‐alanine	144.34 ± 8.91	380.92 ± 5.26
beta‐aminoisobutyric acid	174.03 ± 6.02	421.99 ± 15.16
gamma‐aminobutyric acid	317.47 ± 10.93	775.46 ± 24.19
Total essential amino acids	1783.36	3008.22
Total	16,088.37	31032.78

Abbreviation: ND, not detected.

^a^ Mean ± SD of triplicate values.

* < 0.05.

The importance of proline in pollen stems from its use by bees as an energy source during flight and also as a phagostimulatory compound (Degrandi‐Hoffman et al. 2013). In this respect, polyfloral bee pollen contains higher levels of proline than sunflower pollen, suggesting that this type of pollen may be a better food source for bees. In addition, the amount of proline in pollen and its ratio to total amino acids is an indicator of the freshness of the pollen and whether it has been stored under appropriate storage conditions. This ratio is expected to be below 0.65 (Serra Bonvehí and Escolà Jordà 1997). In our study, the proline/total amino acid ratio was calculated as 0.11 for sunflower pollen and 0.097 for polyfloral bee pollen. These values indicate that both pollen samples were fresh and stored under appropriate conditions.

### 3.6 Vitamin B Profile

Bee pollen is a natural product rich in vitamin content. The vitamin composition of bee pollen, which contains a significant amount of hydrophilic vitamins, varies greatly depending on its plant origin (de Arruda et al. 2013). In this study, the quantitative distribution of B group vitamins in bee pollen was analyzed; according to the results obtained, it was determined that vitamin B9 (folic acid) was the vitamin detected in the highest amount in both monofloral (20.80 mg/kg) and polyfloral (20.77 mg/kg) bee pollen samples (Table 5).

**TABLE 5:** Vitamin B profile of bee pollen samples (mg/kg).

Vitamin B	Monofloral sunflower^a^	Polyfloral^a^
Biotin (B7)	0.22 ± 0.0037	0.25 ± 0.01
Cyanocobalamin (B12)	0.39 ± 0.002	0.39 ± 0.005
Folic Acid (B9)	20.80 ± 0.006	20.77 ± 0.008
Nicotinic acid (B3)	2.31 ± 0.03	0.98 ± 0.008
Pantothenic acid (B5)	1.10 ± 0.09	2.55 ± 0.13
Pyridoxine (B6)	0.18 ± 0.04	0.11 ± 0.14
Riboflavin (B2)	0.15 ± 0.004	0.12 ± 0.002
Thiamine (B1)	9.06 ± 0.01	9.04 ± 0.02
Total	34.21	34.21

^a^ Mean ± SD of triplicate values.

The lowest concentration of vitamin B in monofloral sunflower bee pollen was riboflavin (B2) with 0.15 mg/kg, while in polyfloral bee pollen it was pyridoxine (B6) with 0.12 mg/kg. In addition, nicotinic acid, pyridoxine, riboflavin, and thiamine levels were higher in monofloral bee pollen compared with polyfloral bee pollen, and these differences were statistically significant. However, only pantothenic acid (2.55 mg/kg) level was significantly higher in polyfloral bee pollen than in the monofloral sample. These findings are remarkable when compared to the literature. Campos et al. (2008) reported that vitamin B9 concentration in bee pollen was in the range of 3–10 mg/kg, and this value was higher in both pollen types in our study. Bayram et al. (2021) conducted a study investigating the vitamin B content of monofloral *Rhododendron ponticum* bee pollen. The study identified the presence of 315 μg/100 g of vitamin B1, 735 μg/100 g of vitamin B2, 1940 μg/100 g of vitamin B5, 455 μg/100 g of vitamin B6, 46 μg/100 g of vitamin B7, and 0.86 μg/100 g of vitamin B12. Similarly, Al‐Kahtani (2017) reported vitamin B6 as 7.7 mg/kg, vitamin B9 as 1.3 mg/kg, and vitamin B12 as 0.5 mg/kg in sunflower pollen. These data show that the concentration of vitamin B9 determined in our study is considerably higher than Al‐Kahtani's findings. However, it is seen that higher values were recorded in the said study in terms of vitamins B6 and B12. These results show once again that the vitamin composition of bee pollen may vary depending on plant origin, environmental conditions, and sampling time.

### 3.7 Carotenoid Profile

The results of the analysis performed using LC–MS/MS device to determine carotenoid compounds in bee pollen samples are presented in Table 6. The carotenoid detected in the highest amount in both monofloral bee pollen (773.402 mg/kg) and polyfloral bee pollen (506.018 mg/kg) was lutein. The lowest carotenoid found in monofloral sunflower bee pollen was neoxanthin with 0.883 mg/kg. In the polyfloral bee pollen sample, the carotenoid detected in the lowest amount was zeaxanthin with 0.574 mg/kg. In addition, fucoxanthin (28.61 mg/kg) detected in polyfloral bee pollen was not detected in the monofloral sunflower bee pollen sample. In this study, in addition to the total carotenoid amount, the carotenoid profile was also examined in detail and all carotenoid compounds analyzed were detected in polyfloral bee pollen, while all carotenoids except fucoxanthin were detected in monofloral sunflower bee pollen. The most predominant carotenoid in both samples was lutein, which is in agreement with the findings reported by Mărgăoan et al. (2014) that lutein is the most common carotenoid in bee pollen. Abd Alla and Salem (2020), in their study with bee pollen from different plant sources such as *Helianthus annuus* , *Trifolium alexandrinum* , *Sesamum indicum,* and *Zea mays* , reported that the most abundant pigment compound in *H. annuus* pollen was β‐carotene with a value of 152.97 mg/kg. In contrast, in our study, the amount of β‐carotene in monofloral sunflower bee pollen was determined as 7.88 mg/kg. It is thought that this difference may be due to differences in the methodological approaches used (analytical techniques, extraction conditions), as well as the amounts of carotenoids being affected by environmental factors such as geographical and plant origin. Indeed, in a similar study conducted by Karkar et al. (2021) on chestnut bee pollen samples collected from different locations, it was reported that both the presence and amounts of bioactive compounds varied according to the geographical origin of the samples. In their study, the researchers detected the astaxanthin compound in only one sample at a level of 6.88 mg/kg in relation to the carotenoid profile of the pollen samples. Among other carotenoid compounds, lutein was found in the range of 0.00–33.42 mg/kg, zeaxanthin 0.00–36.38 mg/kg, β‐cryptoxanthin 0.00–44.67 mg/kg, α‐carotene 0.00–152.42 mg/kg, and β‐carotene 4.45–395.02 mg/kg. In contrast, in our study, all‐trans‐astaxanthin was detected only in the monofloral pollen sample and at a lower level, at 1.441 mg/kg. Lutein content, on the other hand, was found at higher levels in both sample groups: measured at 773.402 mg/kg in the monofloral pollen sample and 506.018 mg/kg in the polyfloral sample. When comparing α‐carotene amounts, similar ranges were observed, with 67.944 mg/kg in monofloral pollen and 119.994 mg/kg in polyfloral pollen. Zeaxanthin levels also showed similarity, being detected at 2.233 mg/kg in monofloral pollen and 0.574 mg/kg in polyfloral pollen. These differences may be attributed to the diversity in botanical origins and geographic locations of the pollen samples. Similarly, in a different study, it was reported that the total carotenoid content of pollen samples collected from various botanical sources and seasons ranged from 0.67 to 690.53 mg/kg (Gámbaro et al. 2025).

**TABLE 6:** Carotenoid profile of bee pollen samples (mg/kg).

Carotenoid	Monofloral sunflower^a^	Polyfloral^a^
All‐trans‐astaxanthin	1.441 ± 0.038	1.183 ± 0.096
Alpha‐carotene	67.944 ± 4.298	119.994 ± 1.11
Beta‐carotene	7.88 ± 0.185	8.348 ± 0.343
Fucoxanthin	ND	28.661 ± 0.741
Lutein	773.402 ± 30.36	506.018 ± 6.125
Neoxanthin	0.883 ± 0.031	1.381 ± 0.033
Violaxanthin	0.969 ± 0.08	10.052 ± 0.453
Zeaxanthin	2.233 ± 0.184	0.574 ± 0.034
Total	854.752	676.211

Abbreviation: ND, not detected.

^a^ Mean ± SD of triplicate values.

### 3.8 Sugar Profile

Within the scope of the study, fructose and glucose analyses were performed using the HPLC‐RID system to determine the sugar content in bee pollen samples. According to the results of the analysis, the amount of fructose in monofloral bee pollen was 11.08 g/100 g, and the amount of glucose was 15.22 g/100 g. In the polyfloral bee pollen sample, the fructose content was 13.86 g/100 g, and the glucose content was 15.03 g/100 g (Table 7). These findings are similar to some studies in the literature. For example, Gardana et al. (2018) evaluated the sugar content of bee pollen samples collected from Colombia, Italy, and Spain, and reported fructose 187 mg/kg and glucose 144 mg/kg in samples originating from Colombia; fructose 231 mg/kg and glucose 159 mg/kg in samples originating from Italy; and fructose 171 mg/kg and glucose 141 mg/kg in samples obtained from Spain (Gardana et al. 2018). However, in another study conducted by Starowicz et al. (2021), the amount of fructose in bee pollen was determined as 140,600 mg/kg, and glucose as 115,600 mg/kg. The data obtained in our study are in high agreement with the findings reported by Starowicz et al. (2021) and reveal that the sugar content of bee pollen may show a wide variation depending on the plant origin, geographical region, and sampling time.

**TABLE 7:** Sugar profile of bee pollen samples (g/100 g).

Sugar	Monofloral sunflower^a^	Polyfloral^a^
Fructose	11.08 ± 0.11	13.86 ± 0.09
Glucose	15.22 ± 1.68	15.03 ± 0.96
Total	26.30	28.89

^a^ Mean ± SD of triplicate values.

### 3.9 Element Profile

Due to its high mineral content, bee pollen is considered as a valuable food supplement, and this feature stands out in scientific studies. In this context, the mineral contents of different bee pollen samples were evaluated in our study, and the presence of a total of 18 macro and micro elements was determined. The highest amount of the mineral phosphorus (P) was found in the monofloral bee pollen sample and was measured at the level of 2394.90 mg/kg. On the other hand, the most dominant mineral in the polyfloral bee pollen sample was potassium (K), and it was detected at 3365.97 mg/kg. These results are consistent with the study of Campos et al. (2008), which confirmed the presence of Ca, Cu, Fe, K, Mg, Mn, P, and Zn in bee pollen. Considering the effect of plant origin, statistically significant differences were determined between monofloral and polyfloral bee pollen, especially in terms of K and Ca contents. Ca 1579.13 mg/kg, K 2282.42 mg/kg, Mg 553.5 mg/kg, and P 2394.90 mg/kg were determined in the monofloral bee pollen sample. In polyfloral bee pollen, the same elements were found as Ca 610.37 mg/kg, K 3365.7 mg/kg, Mg 435.8 mg/kg, and P 2145.74 mg/kg, respectively (Table 8).

**TABLE 8:** Mineral profile of bee pollen samples (mg/kg).

Essential elements	Monofloral sunflower^a^	Polyfloral^a^
B	63.107 ± 0.40	10.722 ± 0.04
Ba	4.798 ± 0.07	6.617 ± 0.04
Be	3.219 ± 0.02	2.723 ± 0.004
Bi	6.51 ± 0.13	6.359 ± 0.13
Ca	1579.126 ± 17.72	610.369 ± 6.18
Co	2.725 ± 0.04	2.756 ± 0.04
Cr	3.331 ± 0.17	3.607 ± 0.02
Cu	13.509 ± 0.12	9.381 ± 0.11
Fe	30.626 ± 0.19	67.009 ± 1.24
K	2282.42 ± 11.91	3365.968 ± 19.80
Li	11.633 ± 2.50	9.921 ± 3.60
Mg	553.496 ± 8.71	435.799 ± 7.16
Mn	14.412 ± 0.09	17.004 ± 0.03
Na	31.254 ± 0.03	39.351 ± 0.25
Ni	7.227 ± 0.20	8.829 ± 0.15
P	2394.894 ± 35.25	2145.738 ± 89.43
Se	4.533 ± 0.57	4.539 ± 0.32
Zn	36.051 ± 0.30	16.508 ± 0.15

^a^ Mean ± SD of triplicate values.

Liolios, Tananaki, Kanelis, et al. (2019) examined the elements P, Mg, K, Ca, Na, Cu, Fe, Zn, and Mn in *Helianthus annuus* bee pollen and found 5486 mg/kg (P), respectively, 582 mg/kg (Mg), 2684 mg/kg (K), 1799 mg/kg (Ca), 176 mg/kg (Na), 15 mg/kg (Cu), 32 mg/kg (Fe), 60 mg/kg (Zn), and 20 mg/kg (Mn). The mineral content of monofloral sunflower pollen samples analyzed in our study was found to be lower compared with the values reported by Liolios, Tananaki, Papaioannou, et al. (2019). Similarly, in the study conducted by Asmae et al. (2021) on eight bee pollen samples of different botanical origins, 2.24–22.73 mg/kg for Ca, 91.85–397.22 mg/kg for Na, 17.07–68.86 mg/kg for Fe, 485.37–4685.5 mg/kg for K, 68.73–793.35 mg/kg for Mg, 2.09–7.18 mg/kg for Cu, 15.28–38.83 mg/kg for Zn, 16.43–126.3 mg/kg for Al, and 0.0033–0.0049 mg/kg for Pb were reported. When the polyfloral bee pollen sample analyzed in our study was compared with the ranges reported by Asmae et al. (2021); Ca and Cu levels were found to be higher, and Na content was found to be lower. Other elements, Fe, K, Mg, and Zn, were similar to the ranges reported in the related study.

### 3.10 Fatty Acid Profile

All lipid classes, including neutral lipids such as glycerolipids, galactolipids, sphingolipids, sterols, triacylglycerols, sterol esters, and waxes, have been identified mainly in mature pollen. Although the lipid classes found in pollen are similar to those found in other tissues of plants, there are important differences in lipid composition. These differences lead to the formation of a pollen‐specific lipidome structure (Ischebeck 2016). For honey bees, lipids are primarily involved in energy metabolism, but they are also critical for bee nutrition, development, and reproduction. For example, fatty acids such as oleic acid and palmitic acid make up about 60% of the body fat of adult worker bees, 40% of larvae, and 58% of queen pupae (Manning 2001). Therefore, the fatty acid profile of pollen, one of the main food sources of bees, is of great importance.

In this study, a total of 37 different fatty acid compounds were analyzed in bee pollen samples (Table 9). The fatty acid profiles of monofloral sunflower bee pollen and polyfloral bee pollen samples obtained from bee colonies in the Thrace region were compared. In both pollen samples, the fatty acid detected at the highest rate was palmitic acid with 23.08% in monofloral sunflower pollen and 23.02% in polyfloral pollen. Following palmitic acid, the second highest detected fatty acid was alpha‐linolenic acid. These two fatty acids were found at similar levels in both samples, and there was no significant difference between them. The differences between the pollen samples were based on saturated fatty acids (caproic acid, caprylic acid, lauric acid, eicosanoic acid, behenic acid, and lignoceric acid) and unsaturated fatty acids (oleic acid, linoleic acid, cis‐11‐eicosenoic acid, cis‐11,14‐eicosadienoic acid, cis‐8,11,14‐eicosatrienoic acid, and cis‐11,14,17‐eicosatrienoic acid). In a study conducted in Serbia involving 26 different bee pollen samples, the proportion of sunflower pollen in one of the pollen groups was determined as 75%, and the presence of caprylic acid, oleic acid, linoleic acid, and alpha‐linolenic acid was detected in these samples (Kostić et al. 2017). In the same study, in parallel with our findings, the highest levels of palmitic acid and alpha‐linolenic acid were reported. However, unlike this study, Kostić et al. reported alpha‐linolenic acid as the lowest amount of fatty acid with 7.54% in the sunflower‐dominant pollen sample. In another study by Nicolson and Human (2013), the highest amount of fatty acid detected in sunflower pollen was lauric acid with 33.2%, followed by palmitic acid (22.9%) and alpha‐linolenic acid (20.46%). In the same study, it was determined that more than 60% of the overall fatty acid profile of *Helianthus annuus* pollen and bee pollen consisted of saturated fatty acids. According to the findings obtained in this study, total saturated fatty acids in monofloral sunflower pollen and polyfloral pollen were 43.547% and 47.935%, respectively. When these values are compared with those reported in the literature, they show that the fatty acid profiles of the samples may vary significantly according to plant origin, environmental factors, and geographical characteristics.

**TABLE 9:** Fatty acid profile of bee pollen samples (%).

Compound	Monofloral sunflower^a^	Polyfloral^a^
Butyric acid	ND	ND
Caproic acid	0.23 ± 0.002	ND
Caprylic acid	6.385 ± 0.661	0.101 ± 0.002
Capric acid	0.179 ± 0.01	0.25 ± 0.017
Undecanoic acid	ND	ND
Lauric acid	5.466 ± 0.385	0.503 ± 0.02
Tridecanoic acid	ND	ND
Myristic acid	0.973 ± 0.066	1.356 ± 0.2
Myristoleic acid	ND	ND
Pentadecanoic acid	0.085 ± 0.006	0.118 ± 0.006
cis‐10‐Pentadecenoic acid	ND	ND
Palmitic acid	23.803 ± 1.202	23.016 ± 1.853
Palmitoleic acid	ND	ND
Heptadecanoic acid	0.172 ± 0.009	0.158 ± 0.005
cis‐10‐Heptadecenoic acid	ND	ND
Stearic acid	3.718 ± 0.267	5.355 ± 0.397
Elaidic acid	0.413 ± 0.026	0.535 ± 0.106
Oleic acid	5.359 ± 0.071	10.181 ± 0.847
Linolelaidic acid	ND	ND
Linoleic acid	7.934 ± 0.779	17.265 ± 0.73
gamma‐Linolenic acid	ND	ND
Alpha‐Linolenic acid	21.783 ± 1.753	19.805 ± 1.522
Eicosanoic acid	0.269 ± 0.026	2.177 ± 0.23
cis‐11‐Eicosenoic acid	2.353 ± 0.156	ND
Heneicosanoic acid	ND	ND
cis‐11,14‐Eicosadienoic acid	0.336 ± 0.022	ND
cis‐8,11,14‐Eicosatrienoic acid	0.329 ± 0.022	ND
cis‐5,8,11,14‐Eicosatetraenoic acid	ND	ND
cis‐11,14,17‐Eicosatrienoic acid	ND	0.149 ± 0.019
Behenic acid	ND	0.729 ± 0.06
Erucic acid	ND	ND
cis‐5,8,11,14,17‐Eicosapentaenoic acid (EPA)	ND	ND
Tricosanoic acid	ND	ND
cis‐13,16‐Docosadienoic acid	ND	ND
Lignoceric acid	2.267 ± 0.155	0.251 ± 0.011
Nervonic acid	ND	ND
cis‐4,7,10,13,16,19‐Docosahexaenoic acid (DHA)	ND	ND
SFA	43.55	34.01
USFA	38.50	47.93
PUFA	30.38	37.22
MUFA	8.12	10.71
Total	82.05	81.95

Abbreviations: MUFA, monounsaturated fatty acid; ND, not detected; PUFA, polyunsaturated fatty acid; SFA, saturated fatty acid; USFA, unsaturated fatty acid.

^a^ Mean ± SD of triplicate values.

## 4 Conclusion

Today, the food industry's focus on heavily processed products has increased consumers' interest in natural and healthy alternatives, and the demand for bee products has increased significantly. Therefore, it is of great importance to reveal the physicochemical and phytochemical profiles of bee products in detail and to evaluate their safety for use. In this study, the chemical composition of monofloral and polyfloral bee pollen samples was comparatively analyzed and their potential for use as functional food was evaluated. The findings revealed that both pollen types contain rich bioactive compounds, but the amount and diversity of the content vary greatly depending on the botanical origin. Considering this fact, monofloral pollen offers a more homogeneous, predictable, and standardizable content, making monofloral pollen, especially monofloral pollen from *Helianthus annuus* (sunflower), more suitable for controlled production processes in pharmaceutical, nutraceutical, and functional product development. However, considering that this study was conducted with only one monofloral and one polyfloral bee pollen sample collected from a single geographical region, all the findings obtained should be considered as preliminary. Indeed, the chemical composition of bee pollen can vary significantly depending on various factors such as harvest time, geographical origin, and environmental conditions as well as botanical origin. This increases the importance of future comprehensive studies with a larger number of samples from different regions. Only in this way can more reliable and generalizable data on the bioactive profile and thus standardization of *Helianthus annuus* pollen sample can be obtained.

## Author Contributions

N.Y., Y.C.G.: conceptualization, methodology, investigation, writing – review and editing, project administration, funding acquisition, Y.C.G.: methodology, validation, investigation, formal analysis: N.Y. All authors have read and agreed to the published version of the manuscript.

## Conflicts of Interest

The authors declare no conflicts of interest.

## Data Availability

Data can be made available from the corresponding author upon request.
